# Comparison of postoperative IL-6 and IL-10 levels following Erector Spinae Plane Block (ESPB) and classical Thoracolumbar Interfascial Plane (TLIP) block in a posterior lumbar decompression and stabilization procedure: a randomized controlled trial

**DOI:** 10.1186/s12871-023-01973-w

**Published:** 2023-01-10

**Authors:** Aida Rosita Tantri, Rahmi Rahmi, Arif Hari Martono Marsaban, Darto Satoto, Ahmad Jabir Rahyussalim, Raden Besthadi Sukmono

**Affiliations:** 1 Department of Anesthesiology and Intensive Care, Faculty of Medicine, Universitas Indonesia, Cipto Mangunkusumo National General Hospital, Jakarta, Indonesia; 2 Department of Anesthesiology and Intensive Care, Faculty of Medicine, Universitas Syiah Kuala, Banda Aceh, Indonesia; 3 Department of Orthopedics and Traumatology, Faculty of Medicine, Universitas Indonesia, Cipto Mangunkusumo National General Hospital, Jakarta, Indonesia

**Keywords:** Block, ESPB, Regional anaesthesia, TLIP

## Abstract

**Background and objectives:**

The erector spinae plane block (ESPB) and classical thoracolumbar interfascial plane (TLIP) block can reduce postoperative pain in lumbar surgery. In this study, we compared the efficacy of ESPB and classical TLIP block in providing perioperative analgesia in patients undergoing lumbar posterior decompression and stabilization by comparing postoperative pain, opioid consumption, and IL-6 and IL-10 serum concentrations between ESPB and classical TLIP block.

**Method:**

This was a prospective, double-blinded, randomized controlled trial in tertiary referral hospitals. Forty patients were randomized into two equal groups, each receiving either ESPB or classical TLIP block. The primary outcome was the difference in IL-6 and IL-10 serum concentrations at baseline and 6 h after lumbar posterior decompression and stabilization. The secondary outcome was total opioid consumption and pain score 24 h post-operatively.

**Result:**

There were no significant differences between the ESPB and classical TLIP block groups in pain score, IL-6 and IL-10 concentration change, and total opioid consumption post-operatively. There was a significant difference in the time until the first dose of morphine was needed between the ESPB and classical TLIP block groups (300 min vs. 547.5 min; *p* = 0.002).

**Conclusion:**

ESPB and classical TLIP block performance during lumbar surgery have comparable pain scores, IL-6 and IL-10 concentration differences pre- and post-operation, and total opioid consumption post-operatively. However, classical TLIP block provides a prolonged duration of analgesia.

**Trial registration:**

ClinicalTrials.gov NCT04951024.

## Introduction

Posterior lumbar decompression and stabilization are surgical procedures that alter spinal instability and deformity [1, 2]. Spinal surgery is a procedure that results in severe pain on the first postoperative day [3]. Inadequate perioperative pain management will impact patient recovery, prevent mobilization and rehabilitation, and increase the risk of chronic pain [4].

Postoperative pain during lumbar decompression and posterior stabilization procedures accounts for the management of nociceptive, neuropathic, and inflammatory pain [5, 6] and is related to the surgical stress response [7–10]. It involves the release of proinflammatory cytokines such as interleukins (IL-1, IL-6), tumour necrosis factor-α (TNFα), and anti-inflammatory cytokines (IL-4, IL-10, transforming growth factor (TGF)). Interleukin-6 (IL-6) is the primary and first mediator involved in the induction and regulation of acute-phase protein synthesis and is released by hepatocytes after surgery or trauma [11]. IL-6 is the most relevant marker of tissue damage during surgical procedures and an important and accurate biomarker for pain [11–13]. The balance between proinflammatory and anti-inflammatory cytokines determines the postsurgical immune response, infection, and wound healing through local and systemic effects [14].

Erector spinae plane block (ESPB) and classical thoracolumbar interfascial plane (TLIP) block have been used recently to provide good pain control after posterior decompression and stabilization [15–18].

ESPB inhibits pain transmission in the dorsal of the thoracic and abdominal spinal nerves [19]. ESPB has been reported as part of multimodal analgesia that could significantly reduce opioid consumption and postoperative pain in lumbar spinal decompression surgery [16, 20]. The thoracolumbar interfascial plane (TLIP) block has also been reported as an effective regional anaesthetic technique for lumbar surgery [21]. TLIP blocks inhibit pain transmission on the dorsal ramus of the lumbar nerve [22–24].

The study was conducted to compare the efficacy of ESPB and classical TLIP block in providing perioperative analgesia among patients undergoing lumbar posterior decompression and stabilization. The primary outcome was the difference in IL-6 and IL-10 serum concentrations at baseline and 6 h after lumbar posterior decompression and stabilization, and the secondary outcomes were total opioid consumption and pain score at 24 h post-operatively.

## Materials and methods

This was a prospective, double-blinded randomized controlled trial in two parallel groups conducted in two tertiary referral hospitals. The study protocol was carried out in accordance with relevant guidelines and regulations and was approved by the Ethics Committee of Medical Faculty Universitas Indonesia (KET-99/UN2.F1/ETIK/PPM.00.02/2021) and Universitas Syiah Kuala – Zainoel Abidin Hospital (122/EA/FK-RSUDZA/2021) prior to the study. The study was also registered at ClinicalTrial.gov (NCT04951024; 06/07/2021). Informed consent for study participation was obtained from all patients and/or their legal guardian(s). No violation of the Helsinki Declaration occurred during the informed consent and data acquisition period.

### Participants

Eligible patients were adults aged 18–65 years old, ASA I-III, with body mass index 18.5–27.0 kg/m^2^ who were undergoing elective posterior lumbar decompression and stabilization surgery. Patients who had a history of chronic opioid consumption, coagulation disorder, cognitive disorder, or infection at the injection site were excluded from this study.

### Sample size calculation

The sample size was calculated based on the unpaired numerical analytical research formula. The standard deviation in the unpaired group was the combined standard deviation of the two groups. Type I error was set at 5%, one-way hypothesis so that Za = 1.64. The type II error was set at 10%, and Zb = 1.28. Based on IL-6 (S = 13.1; X1-X2 = 13) and IL-10 (S = 8.6; X1-X2 = 9) concentrations [14] in a previous study, the calculated sample size was as follows:

$$n1=n2=2{\left(\frac{\left(Za+Zb\right)S}{X1-X2}\right)}^{2}$$

Za = standard derivative alpha.

Zb = standard derivative beta.

S = the combined standard deviation of the compared groups.

X1 – X2 = the minimum difference in the mean that is considered significant.

There are a limited number of studies that utilize IL-6 and IL-10 as markers of inflammation, especially in patients undergoing spinal surgery. In this study, we used Amin et al.’s study [14], which has the most similar population, to determine the S value in a study of pro- and anti-inflammatory cytokines in surgery.

This study would require a sample size of 18 for each group. We included 18 participants in each group. A total of 40 participants enrolled in this study, with comparable allocation to two arms, allowing for a drop-out of 10%.

### Procedures

Patients were randomized by computer-generated block randomization (www.randomizer.org) in a 1:1 ratio to the ESPB or classical TLIP block groups with stratification according to centre. Random allocation was performed by a research assistant who was not directly involved in the research. Randomization results were put into opaque sealed envelopes. The patients, anaesthesiologists who performed intraoperative monitoring, and the Acute Pain Service personnel were blinded to the intervention.

The first blood sample for measuring IL-6 and IL-10 levels was taken when the patient arrived in the operating room. An anaesthesiologist who was not aware of the size of the randomization blocks opened the sealed envelope. ESPB or TLIP block was performed according to the subject allocation.

**Fig. 1:**
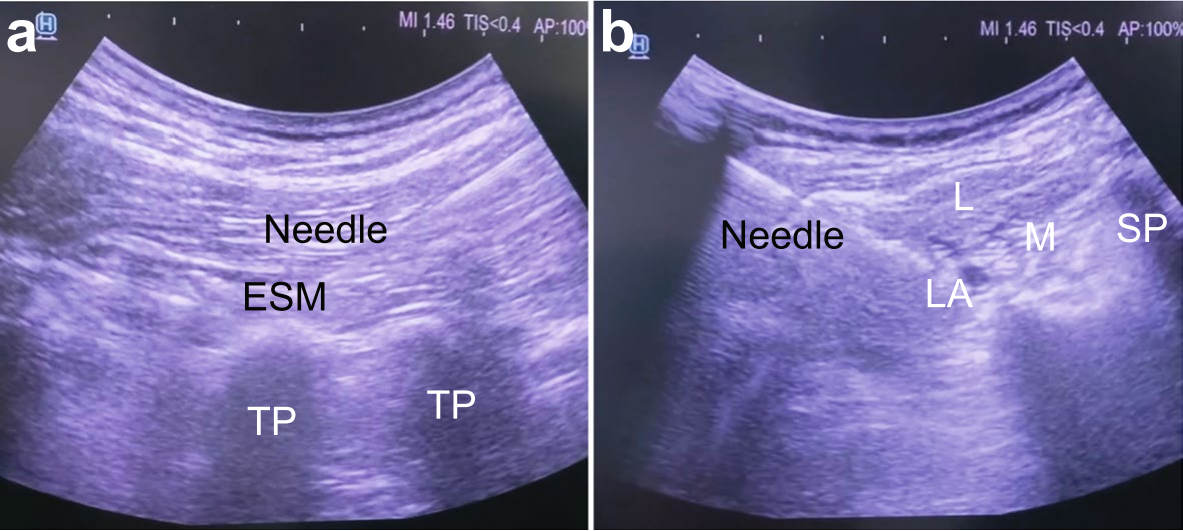
**A** ESPB. **B** Classical TLIP block (L: longissimus, M: multifidus, SP: spinosus processus, LA: local anaesthetic, ESM: erector spinae muscle, TP: transversus processus)

ESPB or classical TLIP blocks were performed at the L3 level in a prone position after the induction of general anaesthesia (Fig. 1). Since both ESPB and TLIP block could extend 2–3 levels cranially and caudally, we provided analgesia for the whole lumbar segment by performing the blocks on L3. ESPB was conducted by local anaesthetic injection into the interfascial space between *m. Erector spinae* and the transverse processes with ultrasound guidance. A low-frequency curved array transducer (Hitachi Arietta® 850 5–1 MHz) was placed longitudinally in a cephalocaudal orientation 2–3 cm lateral to the midline of the L3 vertebral column. After identifying *m. Erector spinae* superficial to the transverse process, an insulated 100 mm 22G nerve block needle was inserted in-plane in the cranial caudal direction until the needle tip contacted the transverse process. Two millilitres of local anaesthetic was injected to confirm proper needle placement deep into the erector spinae. Twenty millilitres of 0.25% bupivacaine was injected bilaterally in 5 ml increments, with aspiration after every 5 ml.

In the classical TLIP block group, the low-frequency curved array transducer was placed in the transverse position at the level of the L3 vertebrae. After the identification of the spinous process, the probe was moved laterally to identify m. multifidus and m. longissimus. An insulated 100-mm 22G nerve block needle was inserted in-plane in the latero-medial direction towards m. multifidus. Two millilitres of local anaesthetic was injected to confirm proper needle placement in the interfascial space between m. multifidus and m. longissimus. Twenty millilitres of 0.25% bupivacaine was injected bilaterally in 5 ml increments, and aspiration was performed every 5 ml.

Another anaesthesiologist, who was blinded to the type of block, entered the operating room after the draping process was completed and performed the intraoperative monitoring. An additional 50 mcg intraoperative fentanyl was given if there was a sudden increase in pulse rate and blood pressure to more than 20% of the basal value. Another 25 mcg of fentanyl was given 5 min later if the pulse rate and blood pressure still escalated.

Postoperatively, paracetamol 1 g/8 h and PCA morphine, with a demand dose of 1 mg, lockout interval of 10 min, and maximum dose of 10 mg/4 h, were given.

In the ward, Acute Pain Service personnel assessed patients’ pain scales and performed blood sample collection. NRS pain scale at 1, 6, 12, and 24 h post-operatively and the first 24 h total morphine consumption were recorded. The second collection for serum IL-6 and IL-10 measurement was taken 6 h post-operatively.

### Statistical analysis

Participants' baseline characteristics were analysed descriptively. Numerical variables are presented as the mean ± SD or median (IQR), while categorical variables are presented as frequency distributions. The data were tested for normality and homogeneity. Data were normally distributed and had a variance of homogeneity if the *p* value > 0.05 in the Shapiro‒Wilk test and Levene’s test, respectively. The difference between the two groups was analysed using an independent t test or Mann‒Whitney test. Two-sided p values were applied with a significance level of 5% for all tests.

## Results

**Fig. 2:**
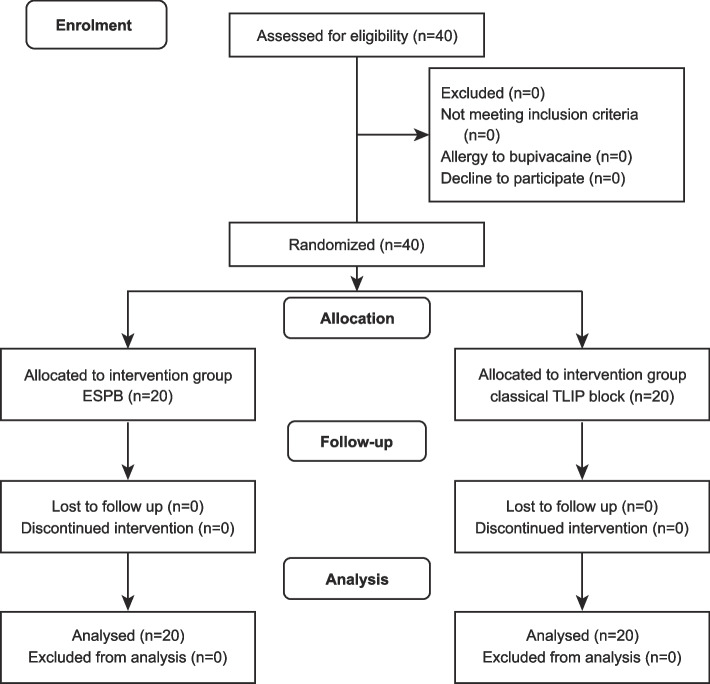
Flowchart of the study

There were 40 eligible subjects recruited, with 20 subjects in each group (Fig. 2). The two groups were comparable regarding age, sex, duration of surgery, and the number of segments involved (Table 1).

**Table 1:** General characteristics of patients

Characteristic	Group	
	ESPB (*n* = 20)	TLIP Block (*n* = 20)
Age (year)	42.05 ± 15.75	47.90 ± 13.4
Sex		
Male [f (%)]	12 (60%)	11 (55%)
Female [f (%)]	8 (40%)	9 (45%)
Body weight (kg)	61.2 ± 8,99	59.3 ± 9,15
Height (m)	1.61 ± 0,07	1.60 ± 0,07
BMI (kg/m^2^)	23.70 ± 4,12	23.0 ± 2,60
Duration of surgery (minute)	145 (86)	180 (104)
ASA physical status		
ASA 1 [f (%)]	1 (5%)	1 (5%)
ASA 2 [f (%)]	11 (55%)	9 (45%)
ASA 3 [f (%)]	8 (40%)	10 (50%)
Number of segments involved		
2 [f (%)]	5 (25%)	5 (25%)
3 [f (%)]	7 (35%)	2 (10%)
4 [f (%)]	3 (15%)	3 (15%)
5 [f (%)]	5 (25%)	10 (50%)
Preblock systolic BP (mmHg)	113.15 ± 11.59	110.90 ± 14.23
Preincision systolic BP (mmHg)	108.20 ± 12.08	102.75 ± 16.51
Preblock diastolic BP (mmHg)	75.55 ± 8.86	75.10 ± 8.58
Preincision diastolic BP (mmHg)	74.10 ± 8.69	73.20 ± 12.02

Numerical variables with a normal distribution are shown as the mean ± standard deviation, numerical variables with an abnormal distribution are shown as the median (interquartile range), and categorical variables are shown as [f (%)]

*ASA* American Society of Anesthesiologists, *BMI* Body mass index, *BP* Blood pressure

There was no significant difference in intraoperative fentanyl consumption (*p* > 0.05), postoperative NRS score, and first 24 h of total morphine consumption (*p* > 0.05) between the two groups. However, the time to first morphine consumption post-operatively in the classical TLIP block group was significantly longer than that in the ESPB group (Table 2).

**Table 2:** Pain score and opioid consumption in the ESPB and TLIP block groups

Variables	Group		*p* value^a^
	ESPB (*n* = 20)	TLIP block (*n* = 20)	
Postoperative NRS			
1 h	3 (2)	3 (2)	0.10
6 h	5 (2)	5.5 (4)	0.44
12 h	3 (1)	3 (1)	0.10
24 h	3 (2)	3.5 (2)	0.54
Intraoperative fentanyl consumption (mcg)	10 ± 20.5	2.5 ± 11.18	0.162^a^
Postoperative 24 h morphine consumption (mg)	10 (5)	7 (3)	0.253^b^
Time to first analgesia (min)	300 (154)	548 (319)	0.002^b^

^a^Unpaired T test

^b^Mann‒Whitney test

Numerical variables with a normal distribution are shown as the mean ± standard deviation, and numerical variables with an abnormal distribution are displayed as the median (interquartile range)

Before induction, the levels of the proinflammatory cytokine IL-6 and the anti-inflammatory cytokine IL-10 were not significantly different between the groups (Table 3). Likewise, 6 h after surgery, the levels of IL-6 and IL-10 were not significantly different (Table 3).

**Table 3:** IL-6 and IL-10 serum concentrations in the ESPB and TLIP block groups

IL-6 Concentration	Group		*p* value^a^
	ESPB (*n* = 20)	TLIP Block (*n* = 20)	
**Interleukin-6 (IL-6)**			
Presurgery	6.30 (10.28)	4.50 (3.55)	0.57
6 h post-surgery	40.36 (61.03)	29.63 (74.05)	0.90
**Interleukin-10 (IL-10)**			
Presurgery	6.01 (5.39)	6.69 (5.0)	0.95
6 h post-surgery	10.54 (4.65)	10.66 (7.79)	0.56

Numerical variables are displayed as the median (interquartile range)

^a^ Mann‒Whitney test

## Discussion

This double-blind, randomized controlled trial (RCT) compares ESPB and classical TLIP block in lumbar posterior decompression and stabilization procedures in terms of stress response cytokines (IL-6 and IL-10), pain scale, and total perioperative opioid consumption. ESPB is effective as analgesia in lumbar spine surgery because it will consistently block the dorsal rami of the lumbar spinal nerves that innervate the back of the vertebra. ESPB also has extensive cranial and caudal spread through the paraspinal muscles from a single injection point, which aids ESPB in covering multiple vertebral levels [25–31]. Meanwhile, TLIP blocks the site of injection, which is located more superficially and further away from the lumbar nerve roots and plexus compared to ESPB. TLIP blocks the dorsal ramus and its branches exclusively in the lumbar distribution.

The local anaesthetic in ESPB was injected deep into the ESP muscle, protecting the solution from being washed out during the surgical procedure and focusing it on post-lumbar surgery pain [27]. Wang et al. found that ESPB provides a better postlumbar spine analgesia profile than TLIP block [27]. ESPB might be better than TLIP block in suppressing the formation of proinflammatory cytokines related to the stress response. ESPB might also provide a better sympathetic and inflammatory response that occurs perioperatively, such as an increase in blood flow, vascular permeability, and leukocyte accumulation.

For decades, cytokines have received more attention for physiological changes after trauma or surgery and acute and chronic inflammation. Under physiological conditions, pro- and anti-inflammatory cytokines act as immunomodulatory elements that prevent excessive damage caused by an inflammatory reaction. A dynamic balance relationship between pro- and anti-inflammatory cytokine changes affects organ dysfunction, immunity, infection, wound healing, and postoperative pain [32].

However, our results differed from those of previous research that hypothesized that a deeper injection of ESPB might provide better analgesia. At six hours, postoperative IL-6 and IL-10 levels were not significantly different between the ESPB and classical TLIP block groups (Table 3). ESPB did not have significantly better perioperative suppression of IL-6 levels compared with TLIP block.

These results may be attributed to pain pathways that were mainly involved in the posterior decompression and stabilization procedure. Pain in lumbar spine surgery mainly involves the dorsal rami of the vertebral nerve, which is covered by both blocks [16, 25], and both blocks work well on the dorsal rami of the vertebral nerve.

Our research results corroborate the previous statement that unlike thoracic ESPB, lumbar ESPB has limited spread to the ventral area. The deep back muscles in the lumbar spine, longissimus, and multifidus are more substantial and prominent in the lumbar region than in the thoracic region. In the lumbar region, local anaesthetic is concentrated within the thick musculature adjacent to the spine [31]. It was postulated that the complex attachments of the deep back musculature to the transverse processes may limit local anaesthetic spread in thoracic ESP injections [33].

Opioid doses that were given during the induction and maintenance of general anaesthesia might also contribute to these results. Opioids might have reduced the degree of perioperative pain in both groups, so the differences were not significant.

Likewise, we also found no significant difference in pain scales at 1, 6, 12, and 24 h post-operatively between ESPB and classical TLIP block (Table 2). The first 24 h of morphine consumption did not differ significantly between the two groups. An additional paracetamol dose of 1 g/8 h that was given as part of postoperative multimodal analgesia might also contribute to these results since the postoperative pain scale might be lowered.

However, we found that the time until first postoperative opioid administration was significantly longer in the classical TLIP block group than in the ESPB group (Table 2). Absorption of local anaesthetics from the injection site depends on the local anaesthetic’s concentration at the site of injection, total dose, vascularization, and concomitant vasoactive that was administered [34–36]. In this study, both groups were injected with identical local anaesthetic doses. In the lumbar region, a median of 5 mL of local anaesthetic is needed to cover one vertebral level by ESPB, which showed that lumbar ESPB has a wider compartment than other blocks. This may be in accordance with the different anatomy of vertebrae and the different spinal curves [25].

The wider compartment of ESPB and more homogeneous distribution of local anaesthetic on the injection plane in the lumbar region led to a rapid and extensive rate of local anaesthetic absorption in ESPB in comparison with other blocks. Cassai et al. reported that peak lidocaine concentration in blood was achieved 5 min after ESPB injection [37]. There have been no papers about local anaesthetic absorption in classical TLIP block yet. In our study, we found that classical TLIP block provides a longer duration of analgesia. Nonetheless, the duration of analgesia of classic TLIP block and ESPB remains inconclusive [27, 38, 39]. Both ESPB and classical TLIP block could provide analgesia 12–24 h after lumbar surgery. Further research regarding the local anaesthetic absorption rate in classic TLIP block is needed.

The limitations of this study included, first, the type of stress response biomarker that had been examined. This study only examined IL-6 and IL-10 cytokine levels to predict the stress response related to the posterior decompression and stabilization procedure. Further research with other mediators and biomarkers could provide more information to make our obtained data more robust. Second, the sample size was calculated using a study in a different setting, which could lead to our study being underpowered.

## Conclusions

There was no significant difference in IL-6 and Il-10 levels and opioid consumption perioperatively between classical TLIP block and ESPB. However, classical TLIP block can provide the same analgesia as ESPB with a longer duration. Both blocks can be used as perioperative analgesia in decompression and posterior stabilization procedures.

## Data Availability

The datasets used and/or analysed during the current study are available from the corresponding author on reasonable request.
